# One-Insertion Stereotactic Brain Biopsy Using In Vivo Optical Guidance—A Case Study

**DOI:** 10.1227/ons.0000000000000722

**Published:** 2023-04-21

**Authors:** Karin Wårdell, Elisabeth Klint, Peter Milos, Johan Richter

**Affiliations:** * Department of Biomedical Engineering, Linköping University, Linköping, Sweden;; ‡ Department of Neurosurgery and Department of Biomedical and Clinical Sciences, Linköping University, Linköping, Sweden

**Keywords:** 5-aminolevulinic acid (5-ALA), Biopsy, Fluorescence, Hemorrhage, Laser Doppler flowmetry (LDF), Microcirculation, Spectroscopy

## Abstract

Stereotactic neurosurgical brain biopsies are afflicted with risks of inconclusive results and hemorrhage. Such complications can necessitate repeated trajectories and prolong surgical time.

**OBJECTIVE:**

To develop and introduce a 1-insertion stereotactic biopsy kit with direct intraoperative optical feedback and to evaluate its applicability in 3 clinical cases.

**METHODS:**

An in-house forward-looking probe with optical fibers was designed to fit the outer cannula of a side-cutting biopsy kit. A small aperture was made at the tip of the outer cannula and the edges aligned with the optical probe inside. Stereotactic biopsies were performed using the Leksell Stereotactic System. Optical signals were measured in millimeter steps along the preplanned trajectory during the insertion. At the region with the highest 5-aminolevulinic acid (5-ALA)–induced fluorescence, the probe was replaced by the inner cannula, and tissue samples were taken. The waiting time for pathology diagnosis was noted.

**RESULTS:**

Measurements took 5 to 10 minutes, and the surgeon received direct visual feedback of intraoperative 5-ALA fluorescence, microcirculation, and tissue gray-whiteness. The 5-ALA fluorescence corroborated with the pathological findings which had waiting times of 45, 50, and 75 minutes. Because only 1 trajectory was required and the patient could be prepared for the end of surgery immediately after sampling, this shortened the total surgical time.

**CONCLUSION:**

A 1-insertion stereotactic biopsy procedure with real-time optical guidance has been presented and successfully evaluated in 3 clinical cases. The method can be modified for frameless navigation and thus has great potential to improve safety and diagnostic yield for both frameless and frame-based neurosurgical biopsy procedures.

## Graphical abstract

**Figure:**
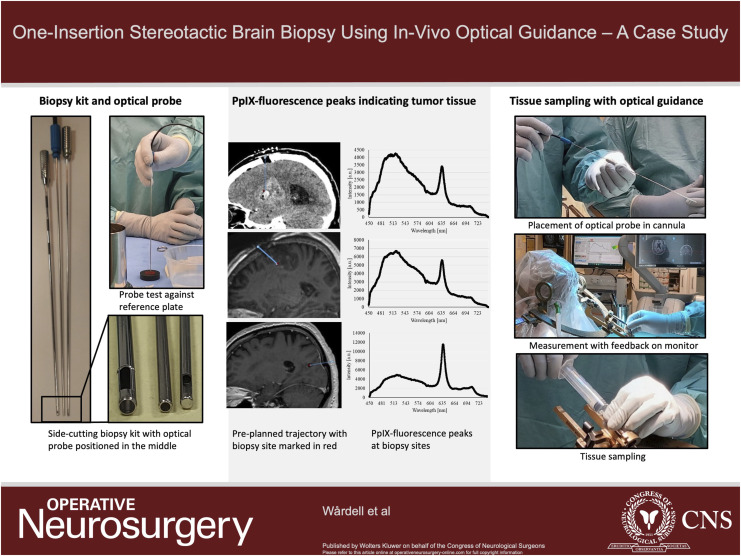

**ABBREVIATIONS:**
a.u.: arbitrary units
FGS: fluorescence-guided surgery
LDF: laser Doppler flowmetry
PpIX: protoporphyrin IX
TLI: total light intensity.

Stereotactic biopsies are afflicted by inconclusive results and complications such as hemorrhage.^1^ Despite meticulous planning, the trajectory can deviate because of brain shift which can result in sampling from the wrong site and inconclusive pathology diagnosis.^2^ It would be beneficial if the sampling error and confirmation bias could be managed because these problems are universal and valid for both stereotactic^3^ and frameless systems.^4,5^ To mitigate complications, intraoperative measurements are required.

Intraoperative methods for neurosurgery have been proposed, of which several are based on optical techniques.^6-9^ Millesi et al demonstrated the clinical benefits with 5-aminolevulinic acid (5-ALA)–induced fluorescence grading during biopsies^2^ in a similar way as during fluorescence-guided surgery (FGS).^10^ Fluorescein^11^ and flow cytometry^12^ are other methods with promising results. We have previously presented an optical probe for combined 5-ALA–induced fluorescence^13^ and microcirculation recording with laser Doppler flowmetry (LDF)^14,15^ during stereotactic biopsies.^16^ Malignant tissue was identified even well outside the area of contrast enhancement in MRI. That concept required 2 instrumental insertions: first the optical probe and then the biopsy needle. To overcome this and possible sources of error, the aim of this study was to present a 1-insertion stereotactic biopsy procedure with optical guidance. It should give real-time feedback to an optimal in vivo biopsy position through 5-ALA–induced fluorescence and act as a “vessel alarm.” The method is exemplified in 3 cases.

## METHODS

### Optical Probe and Biopsy Kit

A probe was constructed with optical fibers along the shaft. The outer dimensions (length = 196 mm, ∅ = 2.1 mm) were designed to fit the cannula of a Sedan Side-Cutting Biopsy Kit 2 (Elekta Instrument AB), which has an opening for the tissue sample of 5 mm with the center 6 mm from the tip. A small aperture was made at the tip of the outer cannula, and the surface was meticulously polished to align perfectly with the optical probe inside. This allowed laser light interaction with the tissue and enabled the biopsy kit and probe to act as a 1-insertion unit with a forward-looking feature compatible with the Leksell Stereotactic System (LSS, G-frame, Elekta Instrument AB).

The optical probe was connected to FluoRa,^17^ an in-house developed system for combined LDF (PF5000, Perimed AB), and fluorescence recording for in situ visual feedback. FluoRa measures tissue microcirculation (perfusion, range 0-1000 arbitrary units [a.u.]), gray-whiteness (total light intensity, TLI, 0-10 a.u.), and 5-ALA–induced fluorescence spectrum (a.u.). After blue-light emission (power = 10 mW, pulse = 400 ms), a protoporphyrin IX (PpIX) peak at 635 nm (red light) is visible in the spectrum in case of malignant tissue. A measurement volume of approximately 1 mm^3^ in front of the probe allows for tissue type identification before samples are retrieved. The LDF features were evaluated in a microsphere solution (PF1001 Motility, Perimed AB) and fluorescence on a reference plate. The biopsy kit and probe were sterilized using Sterrad. The FluoRa system, probe, and modified biopsy kit were used for investigational purpose.

### Patients, Imaging, and Trajectory Planning

Three patients referred for biopsy between June and August 2022 were included. They had suspected brain tumors with locations not eligible for resection, and thus, diagnostics was required before decision on oncological therapy. This study was approved by the local ethics board (EPN 2015-138-32), and all patients gave informed written consent. Approximately 3 hours before surgery, the patients were given an oral dose of 5-ALA (20 mg/kg, Gliolan, Medac GmbH) dissolved in water, the standard dose used for FGS.^18,19^

Stereotactic T_1_-weigthed (w) with gadolinium contrast, T_2_w, and T_2_w fluid-attenuated inversion-recovery (FLAIR) MRI (Ingenia 3T, Philips Healthcare) were acquired on the day of surgery with the LSS. Two trajectories were planned (StealthStation, S8, Medtronic Inc) and biopsy positions defined.

### One-Insertion Stereotactic Biopsy with Optical Guidance

**FIGURE 1.**
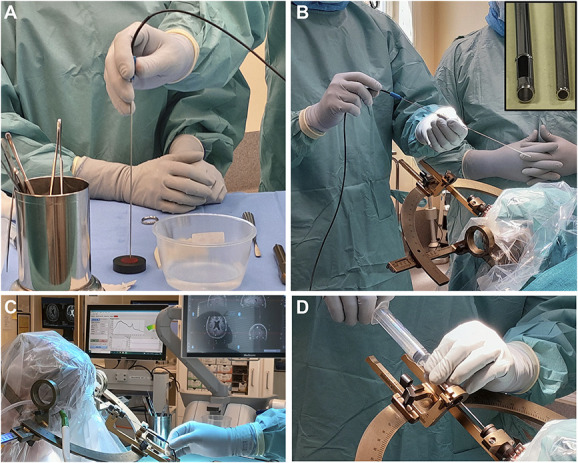
An overview of the measurement procedure. **A**, Test of optical probe and system against a sterile static reference plate. **B**, Insertion of the optical probe into the modified cannula. A close-up of cannula and probe is shown to the right. **C**, Intraoperative measurement. The probe is inserted stepwise, and recorded data are presented in real-time on the screen as seen in the rear. **D**, After withdrawing the optical probe, the biopsy needle is inserted through the cannula and a tissue sample is taken.

The biopsy procedure was fulfilled according to the clinic's routine, with the addition of optical measurements. An overview is photo-documented in Figure 1. First, the optical probe and the system performance were checked against the sterile reference plate (Figure 1A). This was followed by insertion of the probe into the biopsy cannula, which was fixated in the LSS (Figure 1B), and manually pushed forward in millimeter steps toward the preplanned target (Figure 1C). At every site, the cannula was kept still and perfusion and TLI recorded for about 10 seconds followed by capturing of 3 spectra. The results were displayed in real-time (Figure 1C). Next, the needle opening was matched to the tissue region with the largest PpIX peaks and the position locked to keep the cannula in place. Then, the probe was withdrawn and replaced by the inner biopsy needle. Tissue samples were taken (Figure 1D) and sent to a pathologist for intraoperative smear-based section examination. The waiting time from the pathological examination was noted. Samples were also taken for detailed postoperative investigation and definitive diagnosis. Postoperative MRI or computed tomography (CT) was performed to exclude bleedings and to verify trajectory and sampling position through coregistration with preoperative MRI in StealthStation.

### Data Analysis and Statistics

The mean ± SD of perfusion and TLI and the size of the PpIX peaks were calculated. Perfusion >500 a.u. indicating the highest risk of hemorrhage was identified based on previous definition.^14,15^ To keep the photobleaching low, the first recorded spectrum at each site was considered. Perfusion, TLI, and PpIX peaks were plotted against the measurement positions along the respective trajectories. Final diagnosis of each biopsy sample was based on Central Nervous System World Health Organization (CNS WHO) 2021.^20^

## RESULTS

**FIGURE 2.**
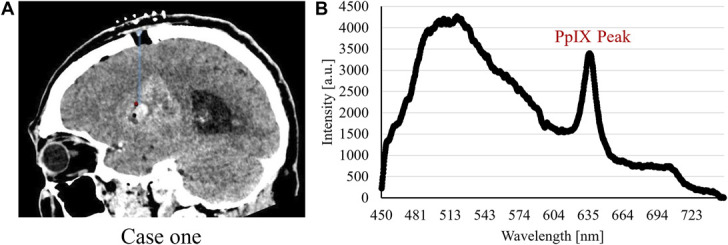
Case 1. **A**, Sagittal coregistered preoperative and postoperative MRI and computed tomography with actual trajectory (blue) and biopsy position (red dot). **B**, The fluorescence spectrum with PpIX peak as captured at the biopsy position. a.u., arbitrary units; PpIX, protoporphyrin IX.

**FIGURE 3.**
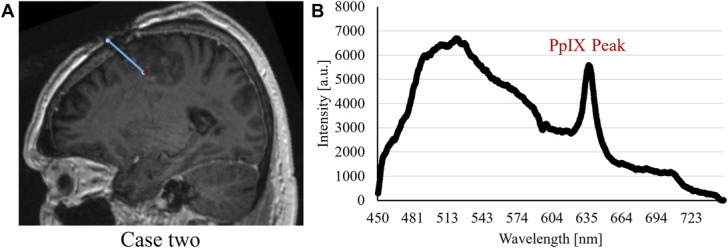
Case 2. **A**, Sagittal coregistered preoperative and postoperative MRI with actual trajectory (blue line) and biopsy position (red dot). **B**, The fluorescence spectrum with PpIX peak as captured at the biopsy position. a.u., arbitrary units; PpIX, protoporphyrin IX.

**FIGURE 4.**
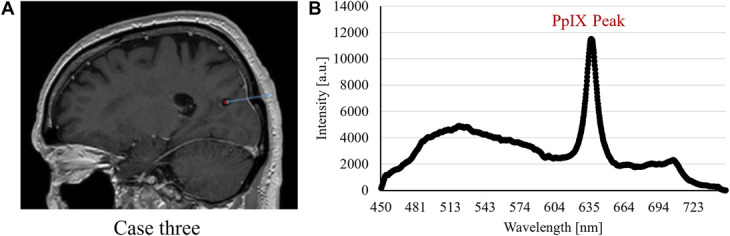
Case 3. **A**, Sagittal coregistered preoperative and postoperative MRI with actual trajectory (blue line) and biopsy position (red dot). **B**, The fluorescence spectrum with PpIX peak as captured at the biopsy position. a.u., arbitrary units; PpIX, protoporphyrin IX.

**FIGURE 5.**
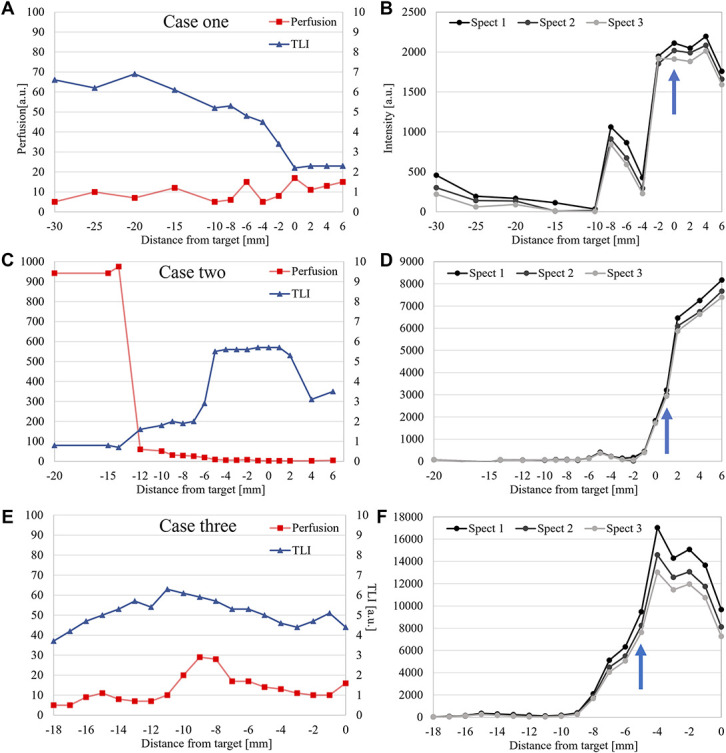
Laser Doppler flowmetry and fluorescence data plotted against the measurement sites along the 3 trajectories. **A**, **C**, and **E**, Perfusion, ie, microvascular blood flow and TLI related to tissue gray-whiteness. **B**, **D**, and **F**, PpIX peak size for the 3 measurements at each position. Spect1, Spect2, and Spect3 represent calculated PpIX peak size from the 3 spectra at the respective measurement position. It is clearly seen that the photobleaching reduces the peak size. Biopsy positions are marked with arrows. a.u., arbitrary units; PpIX, protoporphyrin IX; TLI, total light intensity.

The optical guidance provided the surgeon with direct feedback of intraoperative fluorescence, microcirculation, and tissue gray-whiteness status. This shortened the surgical time because only 1 trajectory was required in the procedures. Depending on the length of the trajectory, the respective measurements took 5 to 10 minutes including documentation. Preparations for closing the skin incisions were performed during the waiting time for the intraoperative pathological results which in all 3 cases corroborated the fluoroscopic PpIX peak findings. Fluorescence spectra with PpIX peaks from the respective biopsy positions are presented in Figures 2, 3, and 4 together with sagittal images presenting the trajectories and sites for biopsy. A summary of recorded data for the patients is presented in Figure 5. It shows the perfusion, TLI, and PpIX peak size along the respective trajectories. The cases are briefly described below.

### Case 1

A 68-year-old man with a history of prostate cancer. Initial presentation of neurological symptoms with focal epileptic seizure and right arm palsy. MRI findings of 2 to 3 partially confluent intracerebral lesions in the left frontal lobe presenting scarce contrast enhancement and moderate perifocal edema. Owing to the uncertain character of the lesions, observation was chosen initially, but after 4 months, distinct progress was noted, and a biopsy was indicated.

Optical measurements were performed in 5 mm steps from −30 to −10 mm and 2 mm steps to +6 mm. Low perfusion was found in all sites, and TLI changed from brighter to darker tissue (Figure 5A). PpIX peaks were visible from −8 to +6 mm (Figure 5B). Tissue was sampled at 0 mm. A bleeding was noted when taking the biopsy. CT confirmed a small bleeding inside the tumor at the biopsy site. The optical finding of suspected tumor was confirmed by the pathologist after 45 minutes. Coregistration verified trajectory and sampling position. Final diagnosis was glioblastoma IDH wild-type CNS WHO grade 4.

### Case 2

A 45-year-old man with known neurofibromatosis I and an intracerebral tumor in the vicinity of the basal ganglia in the right hemisphere, without any signs of progress over the last years. Now apparent growth and expansive effect on central structures, hence indicated for a biopsy.

LDF showed perfusion >500 a.u. at the 3 first sites (Figure 5C). It was confirmed that the biopsy kit with the probe inside was not completely fixed to the LSS and thus still hovering in the cortex. The perfusion decreased when the biopsy kit with the probe was appropriately inserted subcortically. From −10 to + 2 mm, measurements were performed in 1 mm steps. The TLI signal increased before PpIX peaks were seen (Figure 5D). Biopsy was taken at +1 mm. The suspected tumor tissue was confirmed by the pathologist after 50 minutes. Coregistration verified trajectory and sampling position and confirmed that no bleeding occurred. Final diagnosis was glioblastoma IDH wild-type CNS WHO grade 4.

### Case 3

A 68-year-old man who had undergone vitrectomy after which cytometry had shown signs of a lymphoma. Consecutive diagnostics revealed a contrast-enhancing tumorous process in the right occipital lobe, indicative for a burr-hole biopsy. A very small, faint lesion was seen in the FLAIR and T1w images. Nothing on the T2w MRI. The T1w and T2w images were therefore coregistered before planning the trajectory.

Optical measurements were performed every millimeter starting from −18 mm to the precalculated target (0 mm). Both perfusion and TLI were low along the entire trajectory. PpIX peaks were visible from −8 mm (Figure 5E). A biopsy was taken at −5 mm, corresponding to fluoroscopic peaks at the central part of the lesion (Figure 5F). The pathology response was received after 75 minutes and confirmed the optical information. Coregistration verified trajectory and sampling position and confirmed that no bleeding occurred. Final diagnosis was lymphoma.

## DISCUSSION

### Key Results

In this article, an investigational 1-insertion stereotactic biopsy procedure with optical guidance has been presented and successfully evaluated in 3 cases. Because the standard procedure has a risk of inconclusive results and hemorrhage,^1^ there is great potential to improve stereotactic brain biopsies by using optical guidance which can give direct visual feedback.

### Interpretation

Other groups have also suggested solutions to these problems.^6-9,11,21^ Widhalm et al^22^ applied placement of biopsy samples under the blue-light microscope to receive an intraoperative preliminary diagnosis during stereotactic biopsies. Millesi et al^2^ showed that “strong” fluorescence has a high diagnostic rate, shortens the surgical procedure, and decreases the number of total biopsy samples and trajectories. They also question whether intraoperative pathology is necessary when strong fluorescence is detected in the sample because omitting this step would reduce operation time and overall cost. There is, however, a major distinction between those and the here presented fluorescence-based method. In FGS, the grading of the fluorescence is performed by the surgeon's subjective naked eye whereas the probe technique objectifies and quantifies the PpIX fluorescence. Depending on probe design, the spectroscopic system can be used for needle biopsies, on the surface or for deep-seated detection of glioma or lymphoma, ie, the same tumor types as during FGS. Independent of method used, attention must be taken to photobleaching of the tissue. To overcome photobleaching, only 3 spectra per position were captured with FluoRa.^17^ In addition, with 400 ms as exposure time, photobleaching appears and the PpIX peak maximum is slightly reduced (Figure 5B, 5D, 5F). Previous studies have shown that most of the PpIX has bleached out within a minute.^23^ In this study, the recommended FGS 5-ALA dose of 20 mg/kg was used. Previously, we used 5 mg/kg which statistically showed the same outcome.^24^ This indicates that a lower-dose 5-ALA might be enough, which could both decrease costs further and, more importantly, reduce the photosensitivity of the skin. More studies are, however, warranted to verify this.

Hemorrhage can be a severe complication that can prolong surgery and cause cerebral lesions. Mizobuchi et al report up to 10% risk of perioperative hemorrhage. They also state that most bleedings appears during cutting with the biopsy needle,^25^ which we also experienced in 1 case. It is obvious that the needle may damage blood vessels inside or in the vicinity of the tumor, and such bleedings are difficult to avoid, especially because these vessels often are of pathological character. Wilson et al^26^ designed a side-looking optical probe with the purpose to identify vessels at the sampling location of the biopsy. Side-looking optical probes^27^ are more challenging to construct compared with when the optical fibers are placed along the probe shaft. With the approach presented in this article, the brain tissue is thoroughly examined millimeter by millimeter before the tissue is biopsied, and blood vessels crossing the trajectory are identified ahead, in any case before biopsy samples are taken.

### Limitations

Both FGS and the probe technique are not label-free and require administration of 5-ALA. Presently, 5-ALA–induced fluorescence is indicative for high-grade tumors and lymphoma but not low-grade tumors. A practical issue to consider is that the probe must be kept still at each measurement site to record an LDF signal without external movement artifacts. Furthermore, in this study, the biopsy kit was moved along the trajectory manually. Work is ongoing to adapt our mechanical insertion device^14,16,29^ used in previous studies for the dimensions of the 1-insertion biopsy kit. This will increase the precision during the cannula movement also when retracting the biopsy kit and thus adjustment of the needle opening to the tissue region with the highest PpIX peak.

### Generalizability

Many clinics are switching from stereotactic to frameless navigation. Work is, therefore, ongoing to adapt this investigational method to other biopsy systems including frameless navigation.^28^ In principle, any biopsy needle kit can be modified for the 1-insertion procedure and used with our measurement system.^17^ To verify the 1-insertion method's full potential, larger studies are necessary.

## CONCLUSION

A 1-insertion forward-looking optical brain biopsy kit adapted for a stereotactic setting was used in 3 clinical cases. Direct feedback of tissue fluorescence, microcirculation, and variation in gray-whiteness was visualized. High fluorescence peaks verified biopsy positions. The method has shown the potential to increase diagnostic yield, reduce the number of trajectories leading to shorter surgical time, and decrease the risk of complications.

## COMMENTS

Obtaining stereotactic brain biopsies for deep-seated gliomas or lymphoma comes with inherent complication risks and variable diagnostic yield. The authors present a case series of a novel technique for stereotactic brain biopsy using an in-house forward-looking probe with optical guidance that enables users to insert a cannula 1 time to obtain both tissue measurements and sampling. The key difference between the equipment used for this study and that used in the authorʼs prior study is the ability to avoid reinsertion of a probe, which theoretically can help mitigate tissue and vessel damage, improve diagnostic yield, and shorten operative time. The authors fashioned a probe with optical fibers to fit the outer cannula of a side-cutting biopsy kit. In regions with high 5-ALA fluorescence, the probe was replaced by the inner cannula for tissue collection. Through 3 well-documented cases, this device demonstrates the potential to reduce complication risk, increase diagnostic yield, and reduce the number of trajectories for brain biopsies. With more refinements, this technology has the potential to also be used in frameless systems and mechanical insertion devices, ultimately leading researchers to further evaluate its safety and efficacy.

**Billy Woodall**

**Jonathan H. Sherman**

*Martinsburg, West Virginia, USA*
